# Profiling of Sub-Lethal in Vitro Effects of Multi-Walled Carbon Nanotubes Reveals Changes in Chemokines and Chemokine Receptors

**DOI:** 10.3390/nano11040883

**Published:** 2021-03-30

**Authors:** Sandeep Keshavan, Fernando Torres Andón, Audrey Gallud, Wei Chen, Knut Reinert, Lang Tran, Bengt Fadeel

**Affiliations:** 1 Institute of Environmental Medicine, Karolinska Institute, 171 77 Stockholm, Sweden; sandeep.keshavan@unifr.ch (S.K.); fernando.torres.andon@usc.es (F.T.A.); audrey.gallud@gmail.com (A.G.); 2 IRCCS Istituto Clinico Humanitas, 20089 Rozzano, Milan, Italy; 3 Center for Research in Molecular Medicine & Chronic Diseases, Universidade de Santiago de Compostela, 15782 Santiago de Compostela, Spain; 4 Department of Biology and Biological Engineering, Chalmers University of Technology, 412 96 Göteborg, Sweden; 5 Max Delbrück Center for Molecular Medicine, 10115 Berlin, Germany; chenw@sustech.edu.cn; 6 Department of Biology, Southern University of Science and Technology, Shenzhen 518055, China; 7 Department of Computer Science and Mathematics, Freie Universität Berlin, 14195 Berlin, Germany; Knut.Reinert@fu-berlin.de; 8 Statistics and Toxicology Section, Institute of Occupational Medicine, Edinburgh EH14 4AP, UK; lang.tran@iom-world.org

**Keywords:** multi-walled carbon nanotubes, nanoparticles, chemokines, macrophages, transcriptomics

## Abstract

Engineered nanomaterials are potentially very useful for a variety of applications, but studies are needed to ascertain whether these materials pose a risk to human health. Here, we studied three benchmark nanomaterials (Ag nanoparticles, TiO_2_ nanoparticles, and multi-walled carbon nanotubes, MWCNTs) procured from the nanomaterial repository at the Joint Research Centre of the European Commission. Having established a sub-lethal concentration of these materials using two human cell lines representative of the immune system and the lungs, respectively, we performed RNA sequencing of the macrophage-like cell line after exposure for 6, 12, and 24 h. Downstream analysis of the transcriptomics data revealed significant effects on chemokine signaling pathways. *CCR2* was identified as the most significantly upregulated gene in MWCNT-exposed cells. Using multiplex assays to evaluate cytokine and chemokine secretion, we could show significant effects of MWCNTs on several chemokines, including CCL2, a ligand of CCR2. The results demonstrate the importance of evaluating sub-lethal concentrations of nanomaterials in relevant target cells.

## 1. Introduction

Nanotoxicology is a scientific discipline aimed at assessing the potential adverse effects of engineered nanomaterials (NMs) as well as enabling the safe use of NMs [1]. Nanotoxicology research has made great strides in the past ten to fifteen years, and efforts to pin down the mechanism(s) of NM toxicity may ultimately inform regulatory frameworks with the goal of exploiting nanotechnology in a safe and sustainable manner [2]. However, much attention has been focused on the same basic paradigms, including the so-called oxidative stress paradigm [1,2]. This has certainly provided considerable insight into the biological and toxicological effects of NMs, but there may not be a one-size-fits-all mechanism with which to explain NM toxicity in different cells or tissues. The use of global omics-based approaches enables the exploration more broadly of biological mechanisms that influence the toxicity and efficacy of NMs [3]. Considerable progress has been made in the last few years in terms of applying transcriptomics and proteomics coupled with computational analysis to address the NM effects [4].

The nanomaterial repository of the Joint Research Centre (JRC) of the European Commission (EC) provides a collection of exhaustively characterized NMs that have been applied as benchmark materials in a number of projects, including the large, EC-funded FP7-NANOREG project, a pan-European project aimed at a common approach to the regulatory testing of nanomaterials [5]. Hence, in FP7-NANOREG, we performed cytotoxicity screening and cytokine profiling of nineteen NMs using the human monocyte-like THP-1 cell line and obtained evidence that diverse NMs can be grouped based on their pro-inflammatory potential [6]. Similarly, in the EC-funded FP7-MARINA project on hazard assessment and risk management of NMs, a comprehensive study was performed on a panel of metal oxides from the JRC repository [titanium dioxide (TiO_2_) (NM103 and NM104), zinc oxide (ZnO) (NM110 and NM111) and silicon dioxide (SiO_2_) (NM200 and NM203)] using a range of cellular assays representing different target organs or systems (immune system, respiratory system, gastrointestinal system, reproductive organs, kidney and embryonic tissues) [7]. The results enabled a hazard ranking of the NMs. The study also revealed cell type-specific response to NMs; overall, the most sensitive cells studied were the murine alveolar macrophages (MH-S). In the EC-funded FP7-ENPRA project, which also focused on hazard assessment of NMs, a panel of NMs procured from the JRC repository were investigated using the human hepatoblastoma C3A cell line, and silver (Ag) and ZnO were found to be more potent with respect to cytotoxicity and cytokine secretion, whereas the multi-walled carbon nanotubes (MWCNTs) and TiO_2_ displayed less toxicity [8]. The conclusion that the effects of NMs are cell type-dependent was also demonstrated in a study of 23 NMs using a panel of ten different cell lines [9]. In fact, even when assessing NM toxicity towards cells originating from the same organ, the outcome was dependent on the specific cell line used, and its origin (human or mouse), as illustrated by the fact that the three lung epithelium-derived cell lines used differed in terms of oxidative stress [9].

CNTs have received particular attention due to their apparent similarities with other fiber-like materials, although inadequate or limited evidence of carcinogenicity exists for most CNTs [10]. Notwithstanding, in a recent study of seven different CNTs and two carbon nanofibers (CNFs), all the tested materials except one highly aggregated MWCNT sample induced genotoxicity in human bronchial epithelial cells (BEAS-2B) [11]. There was a tendency for CNTs/CNFs with increasing length and diameter to display slightly greater toxicity. Di Cristo et al. [12] performed a comparative study of the toxicity of three benchmark MWCNTs obtained from the JRC repository (NM400, NM401, and NM402) using two murine macrophage cell lines (RAW264.7 and MH-S) and found that long and needle-like NM401, but not short and tangled NM400 or NM402, affected cell viability in a dose-dependent manner in both cell models. For this reason, we selected NM401 as representative MWCNTs for our studies. For comparison, we chose Ag and TiO_2_, which have been shown in numerous previous studies to possess high and low toxicity potential, respectively. The three NMs [Ag (NM300K), TiO_2_ (NM104), and MWCNTs (NM401)] were tested using two human cell lines, the lung adenocarcinoma cell line A549 (often used as a model of the lung epithelium) and THP-1. Following cytotoxicity screening using established protocols [6], we performed genome-wide transcriptomics analysis by applying RNA sequencing, coupled with pathway analysis [13,14]. The results were then corroborated by cytokine–chemokine profiling.

## 2. Materials and Methods

### 2.1. Nanomaterials

The NMs used in this study are classified as representative test materials and include a (random) sample from one industrial production batch. This ensures that the sample has been homogenized and is sub-sampled into vials under reproducible (GLP) conditions, and that the stability of the sub-samples is monitored. Thus, to the extent possible for industrial materials, all the sub-samples should be identical and differences in test results between laboratories for the same endpoint should not be due to differences in the NMs tested [15]. For detailed physicochemical characterization of the selected NMs [Ag (NM300K), TiO_2_ (NM104), and MWCNTs (NM401)] performed at the JRC, refer to: Rasmussen et al. [15] and references therein. For the dispersion of the NMs, the SOP developed under the EC-funded NANOGENOTOX joint action was used, as described previously [6]. Briefly, stock dispersions were prepared by pre-wetting the NM powders in 0.5 vol% ethanol followed by dispersion in sterile-filtered 0.05% w/v bovine serum albumin (BSA)-water (Milli-Q^®^ water, Sigma-Aldrich, Stockholm, Sweden). Both the water and the BSA (obtained from Sigma-Aldrich, Stockholm, Sweden) were endotoxin-free. The samples were then dispersed by sonication (16 min at 400 W) before being added to cell cultures.

### 2.2. Human Cell Lines

Human THP-1 acute monocytic leukemia cells and A549 lung adenocarcinoma cells were obtained from the American Type Culture Collection (ATCC) (Manassas, VA, USA). The cells were mycoplasma tested regularly using MycoAlert^®^ mycoplasma detection kit (Lonza, Basel, Switzerland). THP-1 cells were maintained in RPMI-1640 medium (Sigma-Aldrich, Stockholm, Sweden), supplemented with 10% heat-inactivated fetal bovine serum (FBS), 2 mM L-glutamine, 1 mM Na-pyruvate, 5.0 × 10^−5^ M β-mercaptoethanol, 100 U/mL penicillin, and 100 mg/mL streptomycin. A549 cells were cultured in DMEM (ThermoFisher, Stockholm, Sweden), supplemented with 10% heat activated FBS, 2 mM L-glutamine, 1 mM Na-pyruvate, 100 U/mL penicillin, and 100 mg/mL streptomycin. To induce differentiation into macrophage-like cells, THP-1 cells were stimulated for 3 days with 150 nM phorbol myristate acetate (PMA) (Sigma-Aldrich, Stockholm, Sweden).

### 2.3. Endotoxin Testing

NMs were tested for endotoxin contamination using the LAL test (Limulus Amebocyte Lysate Endochrome, Charles River Endosafe, Charleston, SC, USA) according to the manufacturer’s instructions. The NMs were all endotoxin-free (<0.5 EU/mL) (data not shown).

### 2.4. Cell Viability Assay

Cell viability was monitored with the lactate dehydrogenase (LDH) release assay using the CytoTox96^®^ non-radioactive cytotoxicity kit (Promega, Stockholm, Sweden), as previously described [16]. The samples were analyzed using the Tecan Infinite^®^ F200 plate reader operating with Magellan v7.2 software (Männedorf, Switzerland). The percentage of cell viability was calculated based on the ratio between the absorbance of each sample and the negative control sample.

### 2.5. Transmission Electron Microscopy

Cellular uptake/localization of NMs was monitored by TEM as described [17]. Briefly, cells were fixed in 2% glutaraldehyde in 0.1 M sodium cacodylate buffer containing 0.1 M sucrose and 3 mM CaCl_2_, pH 7.4. Cells were then washed and post-fixed in 2% osmium tetroxide in 0.07 M sodium cacodylate buffer containing 1.5 mM CaCl_2_, pH 7.4, at 4 °C for 2 h, dehydrated in ethanol followed by acetone, and embedded in LX-112, an epoxy derivative. Sections were contrasted with uranyl acetate followed by lead citrate and were examined in a Tecnai 12 Spirit Bio TWIN TEM (FEI, Eindhoven, The Netherlands) at 100 kV. Digital images were obtained using a Veleta camera (Olympus, GmbH, Münster, Germany).

### 2.6. Cytokine and Chemokine Analysis

TNF-α released in the culture medium was measured using an ELISA kit following the instructions provided by the manufacturer (MabTech, Nacka, Sweden). The absorbance of the reaction product was measured using a spectrophotometer (Tecan Infinite^®^ F200, Männedorf, Switzerland) and the results for each sample were calculated using a standard curve of recombinant human TNF-α protein. Lipopolysaccharide (LPS) (100 ng/mL; Sigma-Aldrich, Stockholm, Sweden) was used as a positive control for TNF-α release. Results are expressed as ng/mL of released cytokine, based on three independent experiments. Furthermore, profiling of cytokines and chemokines released by THP-1 cells was performed by using the U-PLEX chemokine panel 1 (human) (K15047K-1) and the V-PLEX pro-inflammatory panel 1 (human) (K15049D-1), respectively. We employed the Meso Scale Discovery (MSD) (Rockville, MD, USA) multi-plex electrochemiluminescence (ECL) platform to quantify cell supernatant concentrations of the indicated biomarkers, according to the manufacturer’s instructions. As a positive control, cells were exposed to 0.1 μg/mL LPS (Sigma-Aldrich, Stockholm, Sweden) for 24 h. The samples were analyzed on the MSD Meso SECTOR^®^ S600 instrument and the data were analyzed using MSD Discovery Workbench^®^ software (v. 4.0) (Rockville, MD, USA). Samples with values below the lower limit of detection (defined as 2.5 S.D. above the background) were excluded from further analysis. The cytokine and chemokine expression data retrieved from the multi-plex assay were further analyzed using hierarchical clustering analysis, as described previously [6]. Complete linkage and Euclidean distances were employed as metrics to draw association dendrograms between cytokines/chemokines and the different treatment conditions. The cluster analysis and the corresponding heatmaps were prepared using R 3.2 [6].

### 2.7. Western Blotting

For protein detection, cells were harvested and lysed at 4 °C in RIPA buffer [50 mM Tris HCl (pH 7.4), 150 mM NaCl, 1% Triton X-100, 0.25% sodium deoxycholate, 0.1% SDS, 1 mM EDTA] supplemented with protease and phosphatase inhibitors plus 1 mM DTT (Sigma Aldrich, Stockholm, Sweden) as described previously [18]. Thirty µg total protein were loaded into each well of a NuPAGE 4–12% Bis-Tris gradient gel (ThermoFisher, Stockholm, Sweden) and subjected to electrophoresis. The proteins were then transferred to a Hybond low-fluorescent 0.2 µm PVDF membrane (Amersham, Buckinghamshire, UK), blocked for 1 h in Odyssey^®^ Blocking Buffer (PBS) (LI-COR), and stained overnight at 4°C with antibodies against NLRP12 (Abcam, Stockholm, Sweden) and GAPDH (ThermoFisher, Stockholm, Sweden) as loading control. The membranes were then probed with the goat anti-rabbit IgG (H+L) HRP-conjugated antibody (ThermoFisher, Stockholm, Sweden) or the goat anti-mouse IRDye 680RD antibody (LI-COR Biotechnology, Lincoln, NE, USA) and proteins were detected using Clarity™ ECL substrates (BioRad, Hercules, CA, USA) and Super RX-N film (FujiFilm Nordic AB, Stockholm, Sweden), or the LI-COR Odyssey^®^ CLx scanner operating with Odyssey^®^ Image Studio software (LI-COR Biotechnology).

### 2.8. RNA Sequencing

Total RNA was extracted from cells harvested at 0 h, 6 h, 12 h, and 24 h of exposure to NMs (25 µg/mL) using the TRIZOL reagent (Life Technologies, Stockholm, Sweden) according to the manufacturer’s recommendations. Total RNA was quantified by NanoDrop^™^ (NanoDrop Technologies, ThermoScientific, Stockholm, Sweden) and RNA quality was assessed using the Bioanalyzer 2100 (Agilent Technologies, Santa Clara, CA, USA). Three biological replicates of each sample were submitted for RNA sequencing [19]. In brief, the sequencing was performed using 1 μg total RNA following the Illumina^®^ mRNA-Seq library preparation protocol (Illumina, San Diego, CA, USA). To this end, poly(A) RNA was isolated by two rounds of oligo (dT)25 Dynabeads^™^ (Invitrogen, Stockholm, Sweden) purification. Then, the chemically fragmented mRNAs were purified by Agencourt RNAClean XP SPRI beads (Agencourt-Beckman Coulter, Beverly, MA, USA) and converted to first strand cDNA, followed by second strand cDNA synthesis. The paired-end sequencing library was prepared from purified double stranded cDNA using the NEBNext^®^ DNA Library Prep Kit (Illumina, San Diego, CA, USA). The purified ligated product was PCR amplified and the prepared libraries were quantified and quality-assessed and sequenced on the Illumina^®^ HiSeq 2000 platform (Illumina, San Diego, CA, USA).

### 2.9. Pathway Analysis

Canonical pathway analysis was done using the Ingenuity Pathway Analysis (IPA) software (content version 24718999) (Ingenuity Systems, Redwood City, CA, USA). The significance of the pathways was estimated through the curated ingenuity knowledge database using a causal analysis approach [20], complemented by hierarchical cluster analysis, as described in Reference [21]. Significant pathways were filtered by *p*-values < 0.001 and activation z-scores > 2 or >2 (data not shown), representing a significant deactivation or activation, respectively. Data were integrated using hierarchical clustering on quantile-normalized data.

### 2.10. Statistical Analysis

One-way analysis of variance (ANOVA), followed by a Dunnett’s or Sidak’s multiple comparison test analysis was used for the analysis of statistical significance, and *p* < 0.05 was considered significant. For nonparametrically distributed data, the two-tailed Mann-Whitney test was used. Statistical tests were performed using GraphPad Prism 8 (San Diego, CA, USA).

## 3. Results

### 3.1. Characterization of Benchmark Nanomaterials

The test materials [Ag (NM300K), TiO_2_ (NM104), and MWCNTs (NM401)] were obtained from the JRC nanomaterial repository and detailed characterization has been provided by the JRC (refer to Table S1 for an overview and see references therein). The MWCNTs are characterized by their rigid and needle-like appearance (length: 4048 ± 2371 nm; diameter: 67 ± 24 nm). The TiO_2_ NMs are rutile, with an average diameter of 25 nm and have been extensively studied in the FP7-MARINA project [7]. The Ag NMs were provided in a colloidal suspension with a primary particle size of 15 nm. The latter NMs were studied extensively in the FP7-NANOREG project (see, for instance, Bhattacharya et al. [6]).

### 3.2. Cytotoxicity Assessment of Nanomaterials

**Figure 1:**
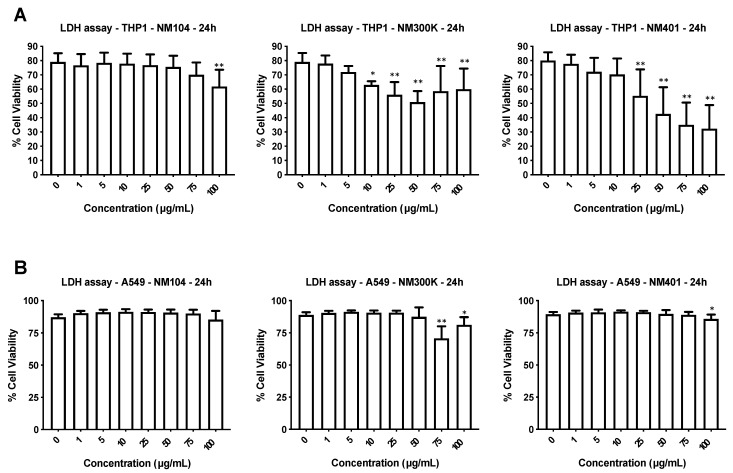
Cytotoxicity screening of representative nanomaterials (NMs). Human macrophage-differentiated THP-1 cells (**A**) and lung epithelium-derived A549 cells (**B**) were exposed to TiO_2_ NMs (NM104), Ag NMs (NM300K), and MWCNTs (NM401) for 24 h and cell viability was evaluated by using the LDH release assay. The results shown are mean values ± S.D. of three independent experiments. * *p* < 0.01; ** *p* < 0.001.

We utilized two human cell lines: the lung cell line A549 and the monocyte-like cell line THP-1. The latter cells were differentiated into macrophage-like cells using PMA [6]. The cells were exposed for 24 h to the three different NMs at concentrations ranging from 1 to 100 µg/mL and cell viability was monitored using the LDH release assay. The TiO_2_ NMs were cytotoxic for THP-1 cells only at the highest dose while Ag NMs and MWCNTs triggered a dose-dependent cytotoxicity at doses above 10 and 25 µg/mL, respectively (Figure 1A). In contrast, the NMs were not cytotoxic towards the A549 lung cell line, apart from a minor effect noted for the Ag NMs at high doses (Figure 1B).

### 3.3. Cellular Uptake of Benchmark Nanomaterials

**Figure 2:**
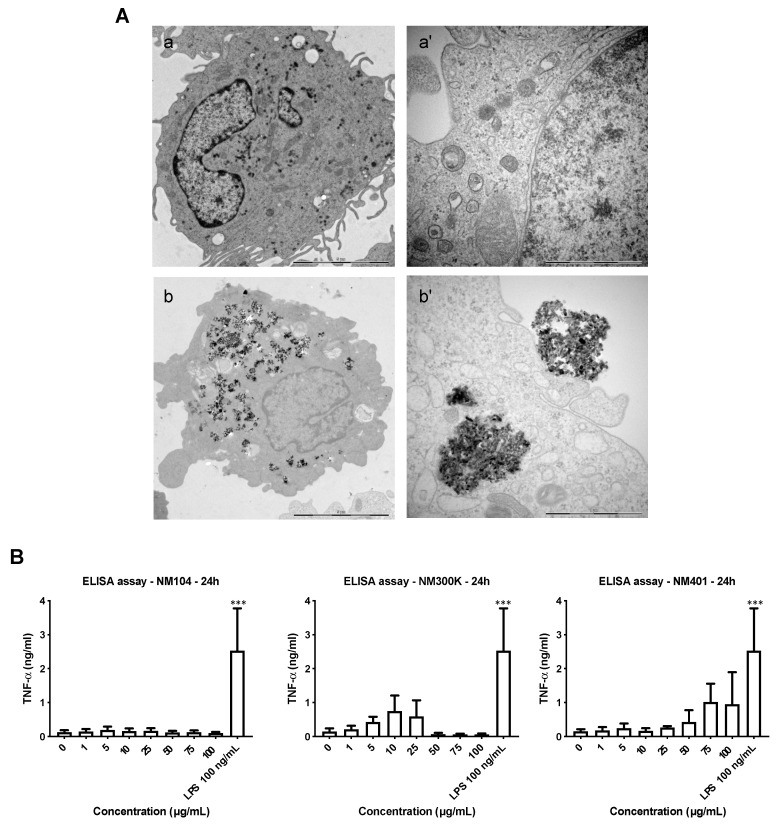
Cellular impact of representative NMs. (**A**) TEM images of untreated THP-1 cells (**a**,**a’**) and cells exposed to TiO_2_ NMs for 24 h at 25 µg/mL (**b**,**b’**). Scale bars: 5 µm (**a**,**b**) and 1 µm (**a’**,**b’**). Refer to Figure S1 for additional findings derived from the TEM imaging. (**B**) THP-1 cells were exposed to TiO_2_ NMs (NM104), Ag NMs (NM300K), and MWCNTs (NM401) for 24 h and TNF-α production was measured by ELISA. The results are mean values ± S.D. of three independent experiments. *** *p* < 0.001.

We monitored cellular uptake by performing TEM imaging of THP-1 cells after exposure for 24 h at 25 µg/mL (a dose at which the cell viability remained >50% for all three NMs). The Ag NMs could not be visualized with certainty at this time-point, possibly due to dissolution of the NMs, as shown in previous studies [22]. The MWCNTs, on the other hand, were found to damage the microtome (Supplementary Figure S1). Therefore, results are shown for TiO_2_ NMs. As seen in Figure 2A, macrophage-like THP-1 cells readily internalized large clusters of NMs in the absence of ultrastructural signs of cell death, consistent with the results of the LDH release assay.

To further explore the cellular impact of the three NMs, we monitored TNF-α production. TNF-α is a prototypic pro-inflammatory cytokine that is strongly induced by LPS. As shown in Figure 2B, while LPS triggered significant secretion of TNF-α, TiO_2_ NMs had no effect, despite the considerable cellular uptake of these NMs. Furthermore, Ag NMs triggered some TNF-α production at low doses, but not at higher doses, whereas the MWCNTs triggered TNF-α production at high doses, albeit less than LPS. These results thus reveal a marginal effect of the NMs on TNF-α production in macrophage-like cells and suggest (indirectly) that these NMs are endotoxin-free, as TNF-α is a potent inducer of LPS [23].

### 3.4. Transcriptomics Analysis of Nanomaterials

**Figure 3:**
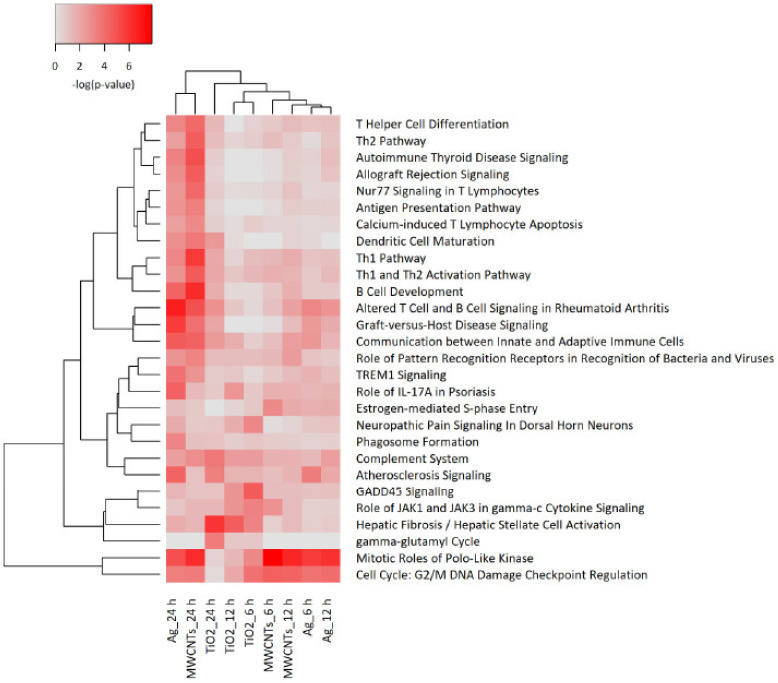
Pathway analysis of transcriptomics data. Macrophage-differentiated THP-1 cells were exposed to TiO_2_ NMs (NM104), Ag NMs (NM300K), and MWCNTs (NM401) for 6 h, 12 h, and 24 h (25 µg/mL) and samples were subjected to RNA sequencing. The heatmap shows the canonical pathway analysis of the transcriptomics data. The significance values indicate the probability of the association of the DEGs with the respective pathway. The cutoff for the *p*-value was *p* < 0.001 for at least one of the conditions.

**Figure 4:**
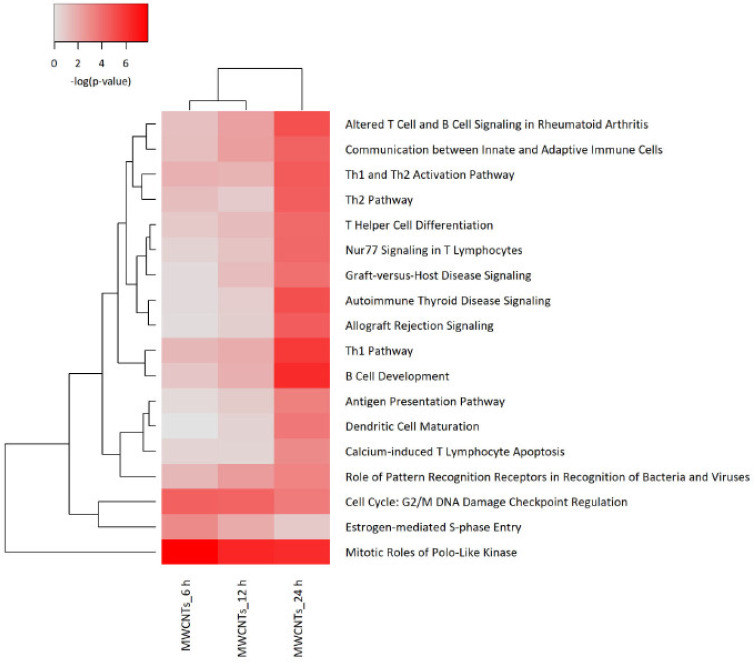
Canonical pathways affected by MWCNTs. The heatmap shows the results of the canonical pathway analysis of the transcriptomics data obtained from THP-1 cells exposed to MWCNTs (NM401) at 25 µg/mL at the indicated time-points. The corresponding analysis for NM401 and NM300K exposed cells is shown in Figures S2 and S3. The significance values indicate the probability of the association of DEGs with the respective pathway. The cutoff for the *p*-value was *p* < 0.001 for at least one of the conditions.

To investigate the effects of the selected NMs in more detail, we applied RNA sequencing. THP-1 cells were selected as a model. The cells were exposed for 6 h, 12 h, and 24 h to NM300K, NM104, and NM401 at 25 µg/mL in order to determine the kinetics of the transcriptomics responses. Samples were sequenced using the Illumina^®^ HiSeq 2000 sequencing platform. RNA sequencing revealed that significant numbers of differentially expressed genes (DEGs) were affected by the NMs (NM300K: 313, NM104: 674; NM401: 124). Only DEGs with a significance level of <0.05 (FDR) and absolute fold-change ≥2 were included in the subsequent analysis. We focused the IPA analysis on immune cells and immune cell lines. The heatmap in Figure 3 shows the hierarchical cluster analysis of the top canonical pathways identified in THP-1 cells exposed to NM300K, NM104, and NM401 at various time-points. The color coding in the heatmap depicts the *p*-values for the pathways shown. The samples corresponding to the 24 h exposure to Ag (NM300K) and MWCNTs (NM401) are grouped together. To further refine the analysis, we analyzed each NM separately. The results for TiO_2_ and Ag are shown in Supplementary Figures S3 and S4, respectively, while the results for MWCNTs are reported in Figure 4. The analysis shows clear time dependence insofar as the changes in gene expression are more robust (based on *p*-values) at 24 h when compared to 6 h and 12 h. We found that cell cycle related pathways were affected by all the tested NMs, and several pathways related to immune cell function were affected by MWCNTs (Figure 4) and Ag NMs (Supplementary Figure S3), but not by TiO_2_ NMs (Supplementary Figure S2). Based on the activation z-scores, cell cycle pathways were deactivated, while immune related pathways were activated (data not shown).

**Figure 5:**
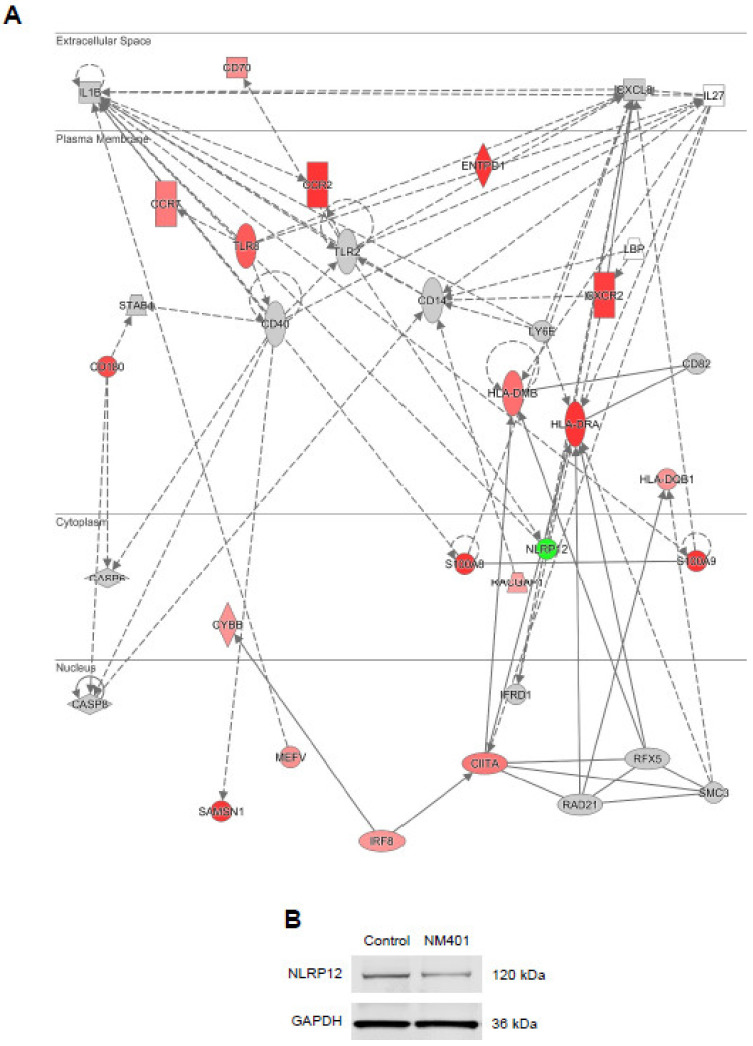
Impact of MWCNTs on the chemokine signaling network in macrophage-like cells. (**A**) The figure depicts the transcriptomics results for MWCNT-exposed (25 µg/mL) THP-1 cells at 24 h. The color coding shows upregulated (red) and downregulated genes (green). Data were analyzed and visualized by using the IPA software tool (Qiagen, Inc., www.qiagenbioinformatics.com/products/ingenuity-pathway-analysis). (**B**) Western blot assay for the expression of NLRP12 in THP-1 cells exposed or not to MWCNTs (25 µg/mL) for 24 h. GAPDH was included as a loading control.

Our analysis showed that the top-most upregulated gene at every time-point in cells exposed to MWCNTs (NM401) was *CCR2*. The log_2_ ratio differential expression values for *CCR2* were 7.938 (6 h), 8.158 (12 h), and 13.712 (24 h). *CCR2* is a chemokine receptor encoding gene, and the corresponding receptor binds chemokine (C-C motif) ligand 2 (CCL2), also referred to as monocyte chemoattractant protein-1 (MCP-1). Figure 5A provides a graphic depiction of the network involving *CCR2* (at 24 h). It is notable that not only *CCR2*, but also *CXCR2* was significantly upregulated in cells exposed to MWCNTs. *CXCR2* is another chemokine receptor encoding gene and the corresponding protein serves as a receptor for CXCL8 (previously known as IL-8). The only downregulated gene in the network was *NLRP12,* encoding a member of the Nod-like receptor (NLR) family of proteins that have been shown to play a role in inflammasome activation [24]. However, *NLRP3* was not affected (data not shown). To validate the RNA sequencing, we checked the expression of the NLRP12 protein in THP-1 cells exposed for 24 h at 25 µg/mL, and we could confirm that the protein expression was decreased following exposure to NM401 compared to the control (Figure 5B).

### 3.5. Cytokine-Chemokine Profiling of Nanomaterials

To further corroborate the transcriptomics results and in order to add the biological context, we performed multiplex assays for the detection of cytokines (IFN-γ, IL-1β, IL-2, IL-4, IL-6, IL-8, IL-10, IL-12p70, IL-13, and TNF-α) and chemokines (Eotaxin, Eotaxin-2, Eotaxin-3, IL-8, IP-10, MCP-1, MCP-2, MCP-3, MCP-4, MDC, MIP-1α, MIP-1β, and TARC). To this end, THP-1 cells were exposed for 24 h to the different NMs (25 µg/mL). LPS (0.1 µg/mL) was included as a positive control. Three independent experiments were conducted and samples with values below the lower limit of detection were excluded from the subsequent analysis. The results confirmed the notion that the NMs did not elicit a pronounced induction of pro-inflammatory cytokines (at 25 µg/mL) (Figure S4). Hence, TNF-α and IL-8 (CXCL8) were not upregulated, while a modest induction was noted for IL-6. NM401 also triggered a modest induction of IL-1β. In contrast, several chemokines were significantly upregulated in response to NM401 (Figure S5). In particular, MCP-1 (CCL2) was upregulated, along with TARC (thymus and activation regulated chemokine, also known as CCL17) and MDC (macrophage derived chemokine, also known as CCL22). It is notable that the magnitude of these responses was similar to that of LPS. NM104 and NM300K, on the other hand, did not show such effects.

**Figure 6:**
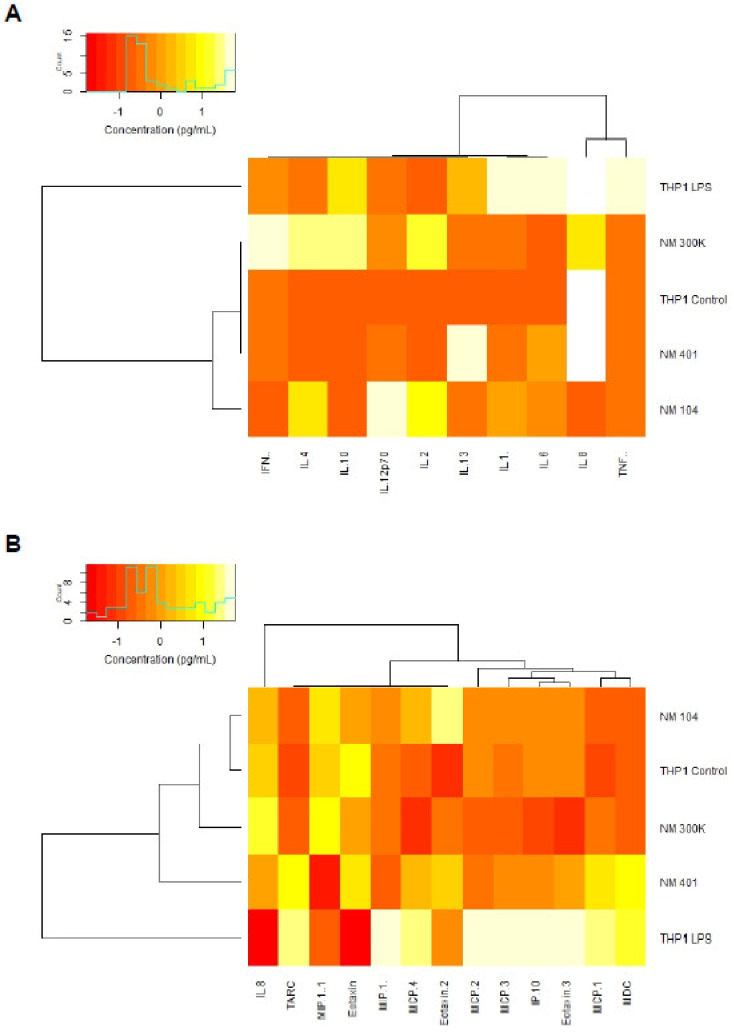
Profiling of cytokine and chemokine production in NM-exposed cells. Macrophage-differentiated THP-1 cells were exposed to TiO_2_ NMs (NM104), Ag NMs (NM300K), and MWCNTs (NM401) for 24 h at 25 µg/mL and cytokine and chemokine production was monitored in the supernatants using multiplex assays. As a positive control, cells were exposed to 0.1 µg/mL LPS. The experiment was performed three times and results for individual biomarkers are reported in Figures S4 and S5. Hierarchical cluster analysis was performed to draw association dendrograms between cytokine (**A**) and chemokine (**B**) responses to NMs. Association clusters for exposures and biomarkers are represented by dendrograms at the **left** and at the **top** of the heatmap, respectively.

To further probe the responses of macrophage-like cells to the three NMs, we performed hierarchical cluster analysis to draw association dendrograms between cytokine and chemokine responses, respectively. LPS exposed samples (supernatants from exposed cells) were identified as separated from the other samples, both with respect to cytokine (Figure 6A) and chemokine responses (Figure 6B). However, the NM401 exposed samples clustered closer to the LPS samples in terms of chemokine responses, whereas NM104, and to some extent, NM300K, segregated with the untreated control samples (Figure 6B).

## 4. Discussion

Using representative NMs from the JRC nanomaterial repository, we have shown that THP-1 cells are more susceptible to NM-induced cell death than A549 cells. The differences between the two cell types could be because epithelial cells are less phagocytic when compared to macrophages. However, we did not perform a quantitative analysis of NM internalization in the present study. It is notable that A549 cells were also found to be refractory to other NMs when compared to primary human lung epithelial cells [25]. Our main finding was that several chemokines and chemokine receptors were significantly affected following a sub-lethal exposure (a dose at which the cell viability remained >50%) of THP-1 cells to MWCNTs. However, we did not observe the induction of pro-inflammatory cytokines, such as TNF-α or IL-8, at the same concentration (25 µg/mL), though MWCNT triggered TNF-α secretion was observed in THP-1 cells when tested at higher doses.

The cytotoxicity of MWCNTs towards THP-1 cells could be related to the shape of these NMs. Indeed, previous investigations have shown that long and needle-like NM401, but not short and tangled NM400 or NM402, elicited a dose-dependent loss of cell viability [12]. Furthermore, in vivo studies have demonstrated that “rod-like” MWCNTs are prone to triggering pulmonary responses with fibrosis and granuloma formation [26,27,28]. However, other properties or features of MWCNTs in addition to their geometric characteristics may also play a role, including the presence of metallic impurities [29,30,31]. We also found that Ag NMs (NM300K) triggered a loss of cell viability in THP-1 cells, while a minor effect was observed in A549 cells at the highest concentrations tested, i.e., 75 and 100 µg/mL. For the latter NMs, cellular uptake and subsequent dissolution of the particles within cells has been identified as one of the key determinants of cytotoxicity [32,33]. Hence, for different NMs, different physicochemical properties may come into play.

It is interesting to consider whether the MWCNTs might emulate some other substrate(s) to which macrophages are programmed to respond. This is presently a matter of conjecture, but we have previously reported that SWCNTs induced chemokine secretion in primary human monocyte-derived macrophages through a Toll-like receptor (TLR)-dependent signaling pathway [16], and a very recent study has provided evidence that the phosphatidylserine (PS) receptor Tim4 (T cell immunoglobulin mucin 4) contributes to the recognition of MWCNTs by murine peritoneal macrophages and plays a role in granuloma formation [34]. Thus, it appears that CNTs might “hijack” pattern recognition receptors that are otherwise deployed by macrophages to respond to microbes or dying cells.

Greco and co-workers recently employed toxicogenomics approaches to study the impact of carbon-based nanomaterials, including MWCNTs on various human cell lines [35,36]. The authors demonstrated that A549 cells are less sensitive than THP-1 cells, as evidenced by the magnitude of the molecular events [35]. In a follow-up study, the authors exposed THP-1 cells to long and rigid MWCNTs and studied genome wide transcription and gene promoter methylation in tandem [36]. Interestingly, among the 220 genes that were found to be affected both at the expression and methylation level, several chemokine encoding genes were identified. In the present study, both CCL17 and CCL22 were significantly upregulated in THP-1 cells exposed to MWCNTs. These chemokines are known to be highly expressed in the thymus and to a lesser extent by dendritic cells and macrophages in secondary lymphoid tissues [37]. Furthermore, both chemokines signal through CCR4, and both have been implicated in type 2 immune responses and were shown to play a role in asthma and in atopic dermatitis [38]. Moreover, we found that CCL2 was significantly induced by MWCNTs. CCL2 is a chemokine, which mediates monocyte chemotaxis and is involved in monocyte infiltration in inflammatory diseases such rheumatoid arthritis [39]. CCL2 acts as a ligand for CCR2, and it is notable that the gene encoding the latter receptor was the most highly upregulated gene in the present study. In a previous in vitro study, we provided evidence that the secretion of CCL3 and CCL5 by primary human monocyte-derived macrophages exposed to endotoxin-free SWCNTs occurred through a TLR2/4-MyD88-NF*κ*B signaling pathway [16]. Furthermore, other investigators have shown that CCR5 (the receptor for CCL3, CCL4 and CCL5) plays an important role in the resolution of pulmonary inflammation in mice exposed to SWCNTs [40]. Snyder-Talkington et al. [41] investigated in vivo responses to MWCNTs by microarray analysis and could show that several chemokine encoding genes were deregulated in the lungs of mice. Using small airway epithelial cells, the authors reported concordant results in vitro with regard to CCL2. Other investigators have shown, using the murine macrophage-like cell line J774A.1, that long (>20 µm) MWCNTs elicited CCL2 (MCP-1) secretion, even at a relatively low concentration [42]. Taken together, single- and multi-walled CNTs prominently affect chemokine signaling in immune-competent cells and especially CCL2 has been highlighted in several studies. The secretion of chemokines such as CCL2 may play a role in the granuloma formation that has been reported in the lungs following pulmonary exposure or in the pleural or abdominal cavity following intra-pleural or intra-peritoneal instillation of MWCNTs [43,44]. Moreover, Sydlik et al. [45] evaluated the biocompatibility of graphene oxide (GO) following implantation in subcutaneous and intraperitoneal tissues in mice and demonstrated a typical “foreign body” reaction (i.e., granuloma formation). The authors found that cells retrieved from these sites secreted significant amounts of monocyte chemotactic protein-1 (MCP-1) (CCL2) and macrophage inflammatory protein-1β (MIP-1β) (CCL4), providing further evidence for a role of these inflammatory chemokines in granulomatous tissue reactions. This also shows that CCL2 (MCP-1) upregulation is not specific for CNTs and is more likely part of a conserved response towards offending pathogens or xenobiotics. Indeed, we previously reported that *CCL2* was upregulated almost 90-fold in the lungs of rats exposed to CuO NMs and these findings were corroborated at the protein level [46]. Moreover, welding-related NMs (essentially, oxides of Fe, Mn and Cr) were found to induce the production of CCL2 in THP-1 cells [47]. Hence, CCL2 may be a particularly sensitive biomarker of immunological perturbations triggered by a variety of NMs.

In addition, we found that *NLRP12* was downregulated in THP-1 cells exposed to MWCNTs, and this was confirmed at the protein level. NLRP12 belongs to the NLR family of proteins involved in inflammasome activation [48]. However, unlike NLRP3, which plays an important role in IL-1β activation in response to a variety of stimuli including MWCNTs [49], NLRP12 seems to attenuate inflammation by dampening NF-κB signaling [50,51]. NLRP12 may also maintain intestinal homeostasis by modulating the gut microbiome [52]. Further studies are needed to investigate the role(s) of NLRP12 for MWCNT-triggered immune responses, and it is worth noting that NLRP12 may impinge on neutrophil function [53]. Neutrophils are a somewhat neglected cell type in nanotoxicology [54].

## 5. Conclusions

Using a combination of transcriptomics approaches and conventional biological assays, we have shown that sub-lethal doses of MWCNTs trigger a deregulation of chemokines and chemokine receptors in a human macrophage-differentiated cell line. These results support the emerging view that CNTs may elicit or interfere with chemokine signaling, and further our understanding of the toxicity of this class of materials [55,56]. However, it is noted that we have only studied one type of MWCNTs, and one cannot extrapolate the findings to other types of single or multi-walled CNTs. Indeed, grouping all CNTs into one material category is scientifically unjustified and may hinder innovation [57,58]. Therefore, further studies are needed to fully address the physicochemical properties that are responsible for the observed biological or toxicological effects of CNTs [10].

## Data Availability

The transcriptomics data were deposited at the NCBI BioProject database (PRJNA286067). Any other results are available from the authors upon reasonable request.

## Supplementary Materials

The following are available online at https://www.mdpi.com/article/10.3390/nano11040883/s1, Table S1: Physicochemical characterization of the selected NMs. Figure S1: TEM imaging of cells exposed to NM401. Figure S2: Pathway analysis of transcriptomics data from cells exposed to NM104. Figure S3: Pathway analysis of transcriptomics data from cells exposed to NM300K. Figure S4: Cytokine release in cells exposed to selected NMs. Figure S5: Chemokine release in cells exposed to selected NMs.
