# Supplementary material for: Profiling of Sub-Lethal in Vitro Effects of Multi-Walled Carbon Nanotubes Reveals Changes in Chemokines and Chemokine Receptors
Source: Nanomaterials (Basel). 2021 Mar 30;11(4):883. doi: 10.3390/nano11040883 (PMC8067081; doi:10.3390/nano11040883)
Supplement: Supplementary file 1 [file nanomaterials-11-00883-s001.pdf]

Table S1. Physicochemical properties of NMs from the JRC nanomaterial repository.

Nanomaterial (NM)	Diameter (TEM) (nm)	Hydrodynamic diameter (DLS) (nm)	Crystalline size (XRD) (nm)	Specific surface area (BET) (m^2/g)	Elemental composition (XPS/ICP-MS)
[1]NM104 (TiO_2)	26 ± 10	128.3 ± 0.8	21–27	41.216	O,C,Ti,Al
[2]NM300K (Ag)	8–50 (average: 17.6)	50-70	NA	NA	NA
[3]NM401 (MWCNT)	60–70 (length: 4048 ± 2371)	NA	10–30	140.46	Na, Fe, Al, Ni, Mg

[1]Rasmussen K. et al. Scientific and Technical Research Reports – Titanium Dioxide, NM-100, NM-101, NM-102, NM-103, NM-104, NM-105: Characterisation and Physico-Chemical Properties. EUR 26637. (2014).

[2]Klein C.L. et al. NM-series of Representative Manufactured Nanomaterials. NM-300 Silver. Characterisation, Stability, Homogeneity. JRC60709. (2011).

[3]Rasmussen K. et al. Scientific and Technical Research Reports – Multi-Walled Carbon Nanotubes, NM-400, NM-401, NM-402, NM-403: Characterisation and Physico-Chemical Properties. EUR 26796. (2014).

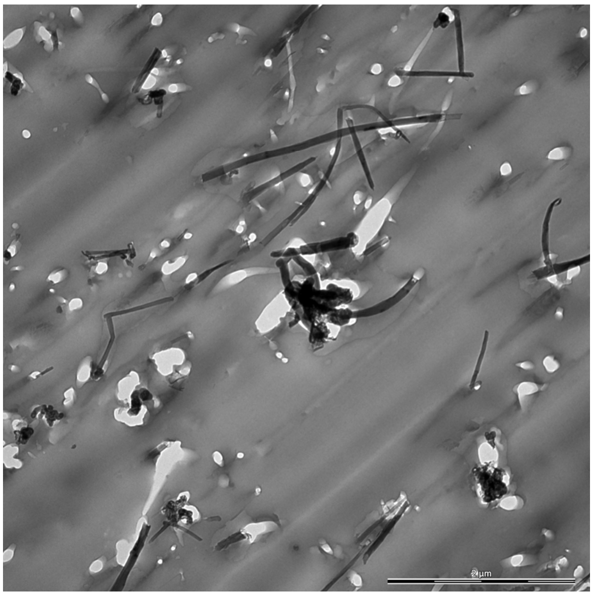

Suppl. Fig. S1. TEM imaging of THP-1 cells exposed to NM401 failed because the microtome blade used to cut the ultrathin sections was damaged by the NM401 material. Nevertheless, some remnants of the rigid, needle-like MWCNTs can be seen.

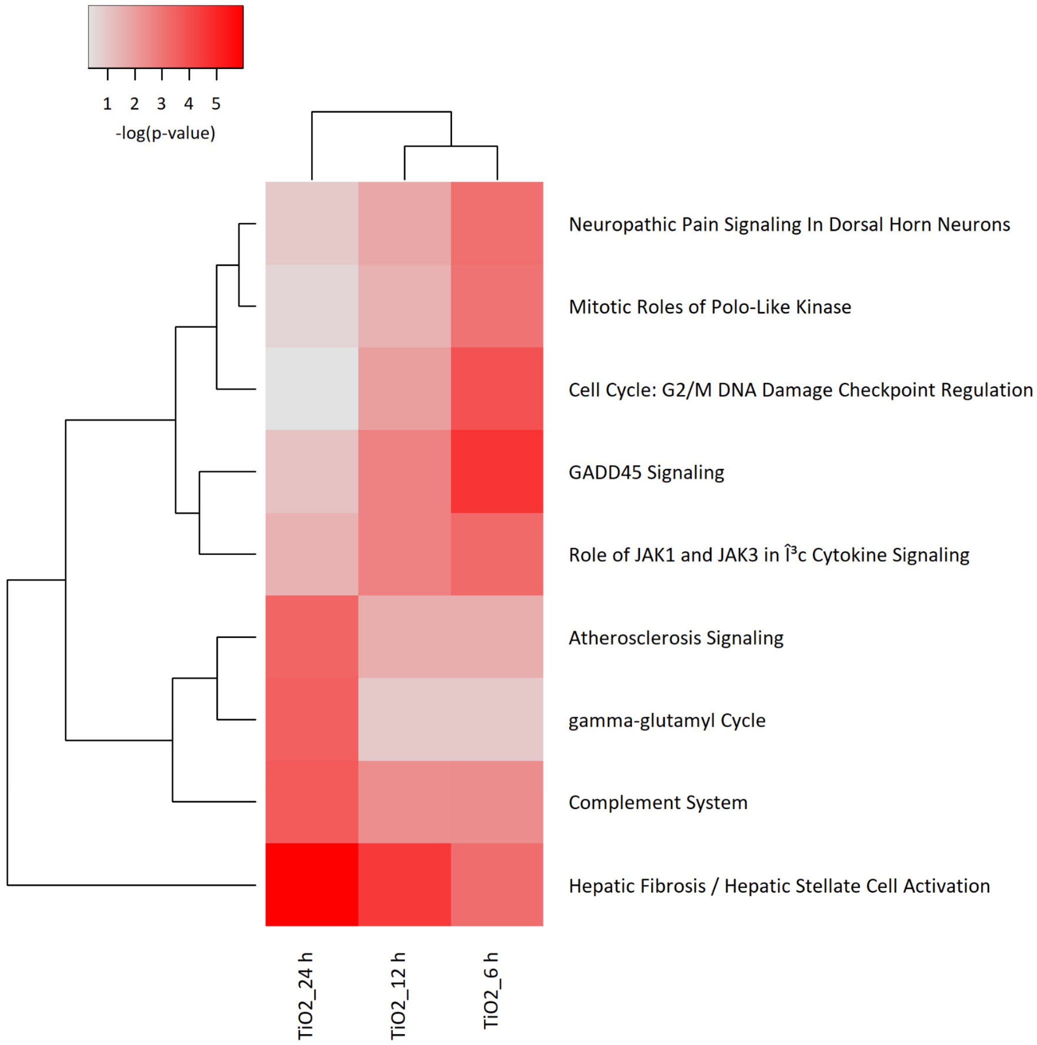

Suppl. Fig. S2. Canonical pathway analysis of transcriptomics data obtained from macrophage-like THP-1 cells exposed to TiO_2 (NM104) at 25 µg/mL. The significance values indicate the probability of association of differentially expressed genes (DEGs) with the respective pathway. The cut-off for the *p*-value was $p<0.001$ for at least one of the conditions.

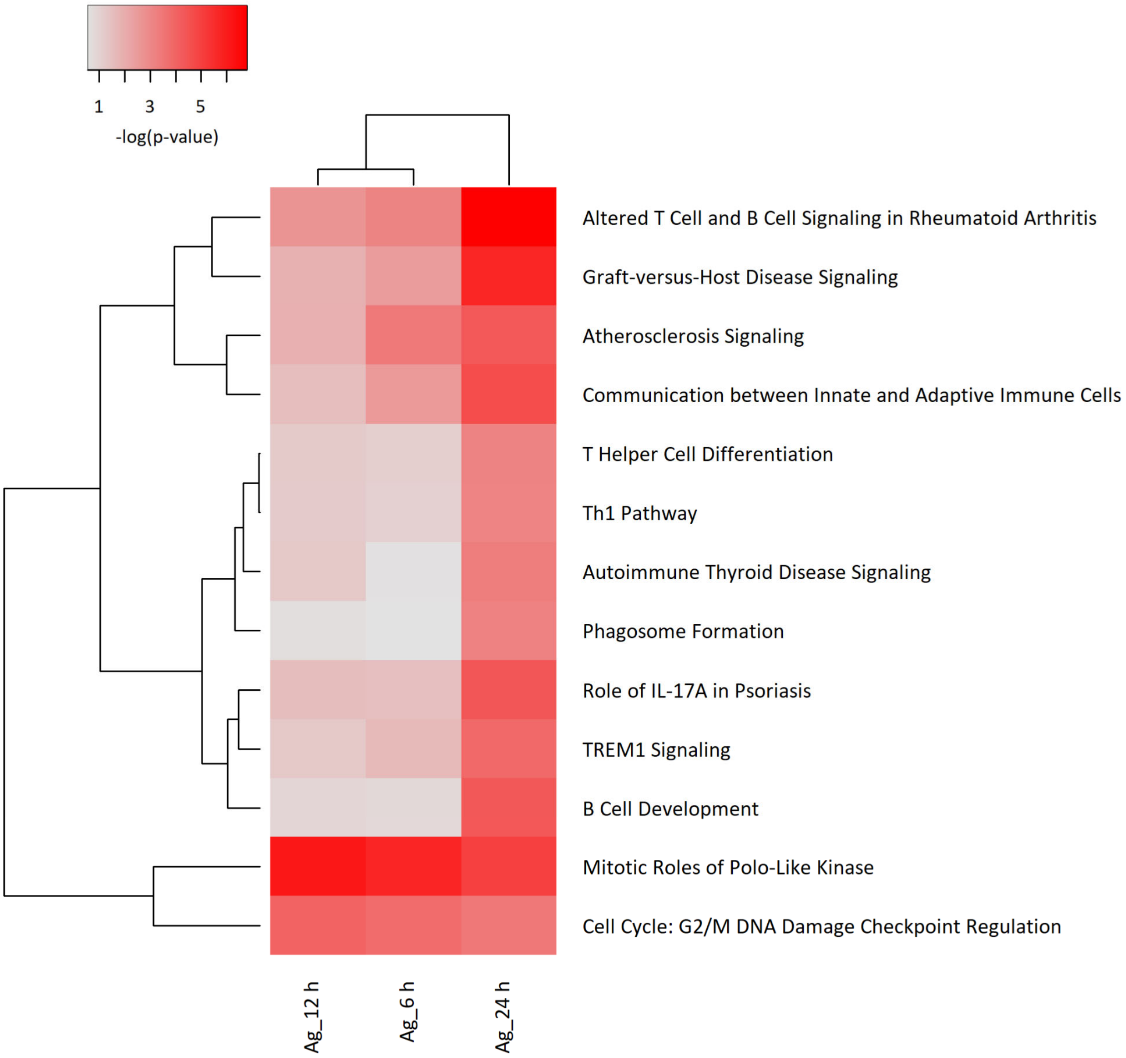

Suppl. Fig. S3. Canonical pathway analysis of transcriptomics data obtained from macrophage-like THP-1 cells exposed to Ag (NM300K) at 25 µg/mL. The significance values indicate the probability of association of differentially expressed genes (DEGs) with the respective pathway. The cut-off for the *p*-value was $p<0.001$ for at least one of the conditions.

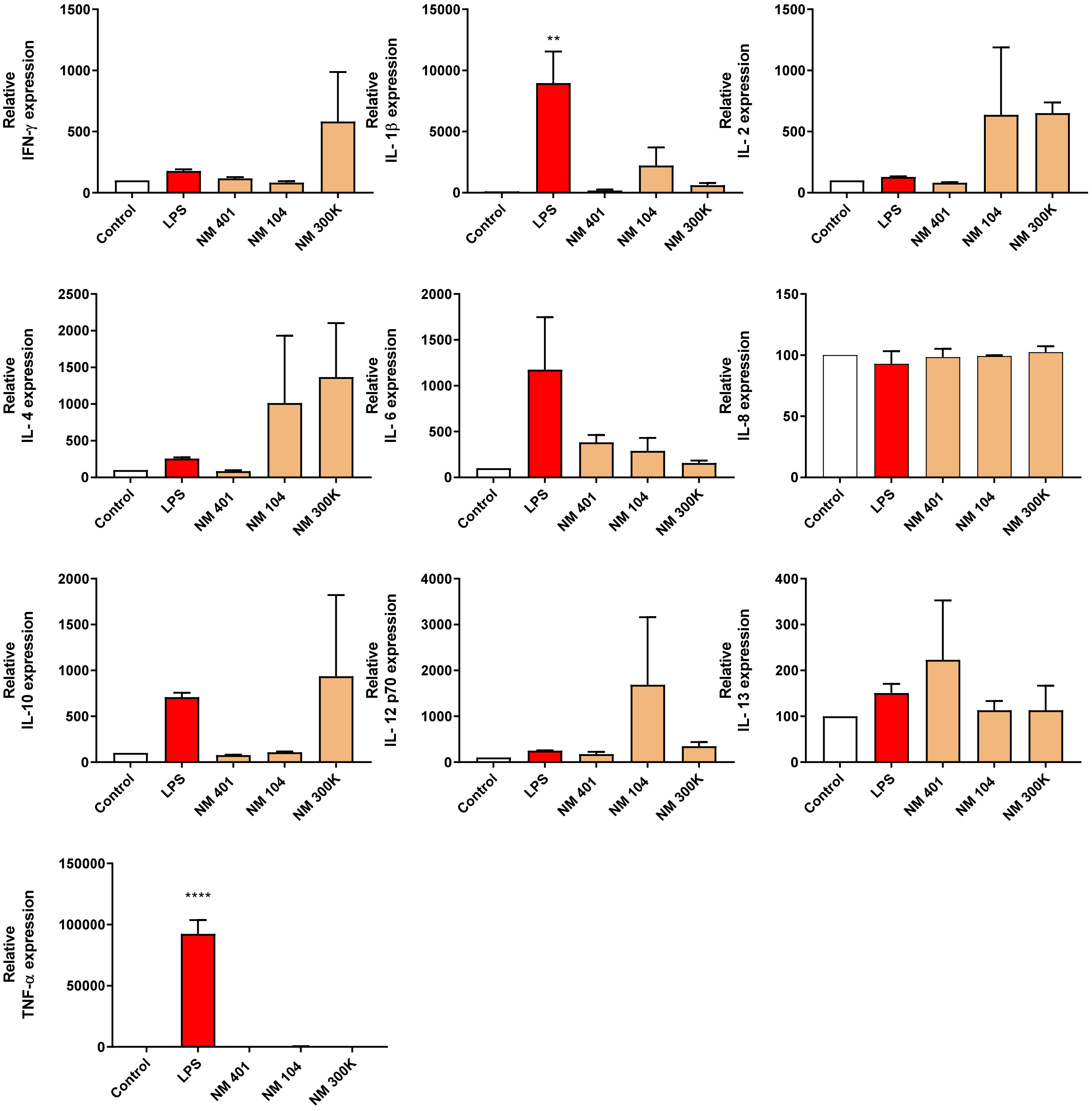

Suppl. Fig. S4. Cytokine release in macrophage-differentiated THP-1 cells exposed for 24 h to NM401, NM104, and NM300K. LPS (0.1 µg/mL) was included as a positive control. The data shown are mean values ± S.D. derived from three independent experiments. *p<0.05, **p<0.01, ***p<0.001, ****p<0.0001 (One-way ANOVA with Dunnett’s multiple comparisons test).

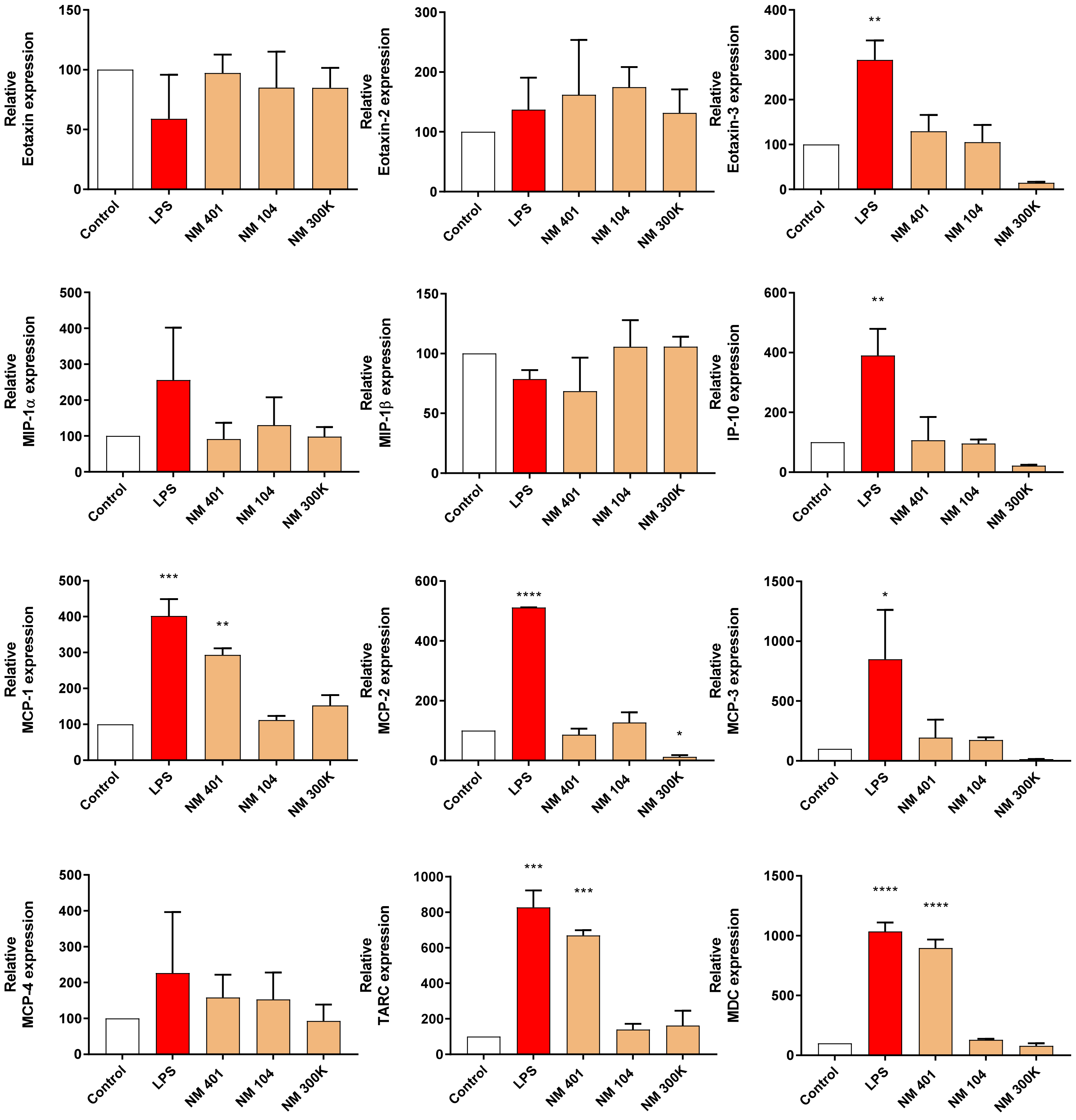

Suppl. Fig. S5. Chemokine release in macrophage-differentiated THP-1 cells exposed to NM401, NM104, and NM300K. LPS (0.1 µg/mL) was included as a positive control. The data shown are mean values ± S.D. derived from three independent experiments. *$p<0.05$, **$p<0.01$, ***$p<0.001$, ****$p<0.0001$ (One-way ANOVA with Dunnett's multiple comparisons test).
